# Psychosocial Distress Due to Interference of Normal Developmental Milestones in AYAs with Cancer

**DOI:** 10.3390/children9030309

**Published:** 2022-02-24

**Authors:** Nelda Itzep, Michael Roth

**Affiliations:** 1 Department of Pediatrics, Section of Pediatric Palliative and Supportive Oncology, University of Texas MD Anderson Cancer Center Children’s Cancer Hospital, Houston, TX 77030, USA; 2 Department of Pediatrics, University of Texas MD Anderson Cancer Center Children’s Cancer Hospital, Houston, TX 77030, USA; mroth1@mdanderson.org

**Keywords:** adolescents, young adults, cancer, psychological pain, psychosocial distress, total pain, posttraumatic growth, resilience

## Abstract

Cancer in the adolescent and young adult phase poses additional challenges to this period of development that is crucial to the transition to independence. This report provides a brief review of normal developmental milestones in this age range while highlighting the disruptive effects of cancer. We focus on the psychological burden of cancer in patients aged 15–39 years and explore the application of the total pain model to highlight the psychological/emotional, social, and spiritual aspects of non-physical pain. We also briefly review posttraumatic growth and resilience. Lastly, we provide a review of areas for possible development and future research.

## 1. Introduction

The adolescent and young adult (AYA) phase is a time fraught with unique challenges, with the ultimate goal of transitioning to independence. Although cancer at any age is life-changing, in the AYA population, it can impair normal development and disrupt this transition to independence. This vulnerable population remains in great need of research to improve short-term and long-term psychosocial outcomes. For this review, the age that defines the AYA population is 15–39 years, as proposed by the National Cancer Institute (NCI) in partnership with the LiveStrong Foundation [1]. While AYAs experience a heavy physical symptom burden secondary to their cancer [2,3] and more treatment-related toxicity compared with younger patients [4,5], they also experience a high psychological burden related to non-physical factors of pain [5]. This will be the focus of this review of the literature.

### 1.1. Incidence, Survival, and Distribution of Disease

Cancer in AYAs represent approximately 5% of all cancer diagnoses, with approximately 90,000 AYAs diagnosed each year in the United States [6]. In total, 6 times more AYAs are diagnosed with cancer compared to children and younger adolescents and recent data shows an increasing incidence in patients aged 15–39 years over time and when compared to patients under 15 years [7]. Survival has improved for AYAs over the past few decades, with the 5-year overall survival approaching 85% [8]; however, outcomes have remained stagnant for many AYA cancer diagnoses, including sarcomas and central nervous system tumors [9]. Cancer types in this group of vulnerable patients also differ when compared to other age groups. For instance, the pediatric landscape of cancer is mostly made up of embryonal tumors and leukemias while that of patients aged over 40 years of age is marked by a higher incidence of lung, urinary, prostate, and colorectal cancers. Meanwhile, within the AYA age range, younger patients are more commonly diagnosed with non-epithelial tumors, such as leukemias, lymphomas, germ cell, and gonadal tumors, while on the older end of the age range, patients are more commonly diagnosed with epithelial tumors, including breast cancer, colorectal carcinoma, and melanomas [10].

### 1.2. Normal Development

**Figure 1:**
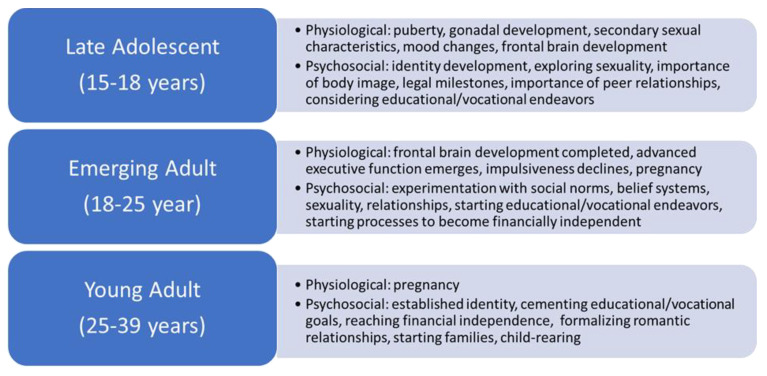
Physiologic and psychosocial aspects of normal developmental stages in AYAs.

To better understand the psychological burden on the AYA cancer population, it is important to note that this age group encompasses a broad range of normal developmental milestones. A brief review of this normal development will provide a framework from which to discern the adverse psychosocial effects of cancer. It is helpful to subdivide this broad group into developmental age groups to better evaluate the physiologic, psychosocial, emotional, sexual, cognitive, vocational, and legal factors that affect these patients. Of note, normal development is fluid and there can be overlap in these groups [11]. See Figure 1 for a summary of these developmental stages.

I. Late Adolescent

Individuals aged 15–18 years of age are in transition between mid and late adolescence [12]. From a physiologic standpoint, adolescents are navigating puberty. Gonadal development is in full swing, and the associated hormonal surges lead to changes in physical appearance and mood [13]. Frontal brain development has begun, but it is not completed until mid-20s [14]. Psychosocially, adolescents are in the midst of developing their own identities and exploring their sexuality [11]. Body image is very important. These AYAs are exploring and setting boundaries from their parents and friends replace their parents as their main source of support [15,16]. At the top of this age range, legal milestones are also achieved, including legal age of voting, smoking, and consent. The driving age, 16 or 18 years, depending on the state in the U.S., is also reached in this period. Many will also complete high school and enter institutions of higher education.

II. Emerging Adult

The emerging adult period includes adults aged 18–25 years as described by Arnett in 2000 [17]. In this age range, advanced executive function/decision-making typically emerges while impulsiveness declines [14]. Frontal brain development is completed near 25 years of age [14]. Socially, young adults are experimenting with social norms, religious or spiritual beliefs, sexuality, and relationships [11]. They are also exploring their career and educational paths [17]. In general, this age group still receives some financial support from their parents or other family, but they are starting to establish paths toward their own financial independence [17]. Additionally, pregnancy is more prevalent in this age group than the late adolescent group.

III. Young Adult

Individuals aged 25–39 years fall into the young adulthood phase, which is a phase of ‘establishment’. Many of these young adults have established their identity and are often cementing their career or educational paths [11]. They may be formalizing relationships or starting families, and many may have reached financial autonomy [18]. 

## 2. Methods

For this narrative review, a comprehensive literature search of peer-reviewed manuscripts was completed using PubMed and Google Scholar. We also reviewed the references of selected manuscripts to identify additional relevant studies.

## 3. Discussion

### 3.1. Concept of ‘Total Pain’ as Applied to AYAs with Cancer

**Figure 2:**
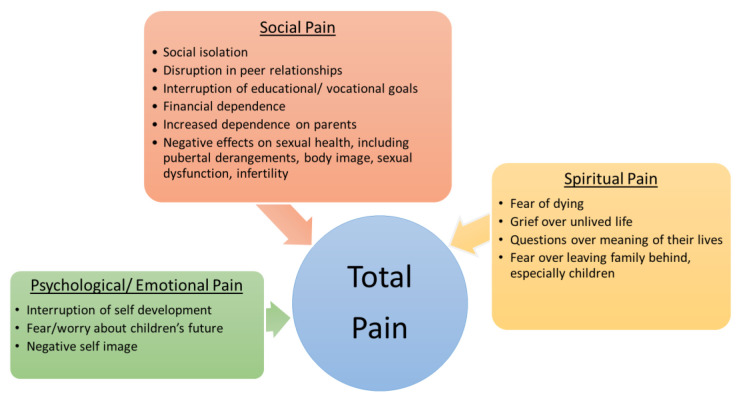
Psychological/emotional, social, and spiritual aspects of total pain in AYAs.

In the 1960s, Dame Cecily Sanders, the founder of modern hospice, described the concept of total pain, which posits that suffering and pain is not just physical but rather it also includes psychological/emotional, social, and spiritual factors [19]. This model can be applied to adolescents and young adults living with cancer. As previously mentioned, they experience physical and non-physical pain. Cancer conflicts with the normal progression of psychological/emotional, social, and spiritual development, thus leading to total pain in this vulnerable group. See Figure 2 for a summary of the psychological/emotional, social, and spiritual aspects of total pain in AYAs.

I. Psychological and Emotional Pain

A diagnosis of cancer is devastating for any person and the psychological strain only adds to the total burden. Psychological pain has been variably defined in the literature. Sandler defined it as the affective state associated with a discrepancy between the ideal and actual perception of self [20] while Orbach et al. described it as “a wide range of subjective experiences characterized as a perception of negative changes in self and its function” [21]. Both definitions have a negative perception of self in common. In AYAs, this is more pointed than ever. Moreover, emotional pain refers to unpleasant feelings related to negative or distressing experiences. Cancer is one such experience, or rather a cluster of experiences, that is particularly taxing emotionally. 

These adolescents and young adults are at the start of their adult lives and striving to become independent productive members of society. They be starting families or already rearing young children. Cancer interrupts this process and, at times, leaves these individuals unable to fulfill social roles, leading to guilt and increased emotional burden [22]. Patients face compromised health and stagnation in their path toward independence [23]. The increased dependence on parents, friends, and/or romantic partners further strains one’s self-identity [22]. Many have experienced good health until their cancer diagnosis and feel the marked difference from their healthy self and their self with cancer [15,22]. AYAs may be starting families or already rearing young children at the time of their cancer diagnosis or during their cancer trajectory. Many will fear and worry about their young children’s future as they confront their mortality. Additionally, they may question the ethics of having children given the possibility of premature death, which further highlights the deviation from this normative role [22].

These feelings of hopelessness, self-disappointment, and decreased self-esteem can lead to significant psychological pain [24] and late effects [25,26]. In a study by McCarthy et al., AYA participants diagnosed with cancer between the ages of 15 and 25 years completed questionnaires within 6–24 months of diagnosis; nearly half reported high rates of general distress and posttraumatic stress symptoms and nearly a third reported symptoms of moderate to severe depression and anxiety [27]. Other studies have also found anxiety and depression to be prevalent in this population [18,28,29] and in AYA cancer survivors [30,31]. 

Yet, timely identification, screening, and management of psychological distress in cancer patients remains variable and the literature is not conclusive. Several reviews looking at studies in the adult population have found that screening for distress has not shown a significant impact on outcomes [32] and do not recommend such screening [33]. In the AYA population, the AYA HOPE study found that mental health was a common unmet service need, and this gap in service was associated with worse health-related quality of life outcomes [34]. Similarly, high levels of posttraumatic stress symptoms and psychological distress are present in the first year after diagnosis, suggesting that screening and interventions should be implemented early in the cancer journey [27,35,36]. However, there is limited data looking at the effectiveness of these screenings and the effect on outcomes.

II. Social Pain

Cancer disrupts nearly every aspect of an AYA patient’s social life. First, social isolation is a known source of distress in this population [22]. Cancer increases dependence on parents and caregivers, thus not allowing for the normal process of separation [15,37]. AYAs are socially, and physically, separated from their peers [23]; often, they cannot attend school or the workplace due to frequent medical appointments, hospitalizations, and diminished physical function. Rather than building relationships, they are increasingly isolated as they focus on cancer treatment [24]. This social isolation can interrupt the formation of peer, romantic, and professional relationships. In the era of social media, AYAs may experience distress as they witness the life they are missing [38] although other studies have suggested that social media can be a powerful tool to promote social/identity development, maintain normalcy, regain control over their life narrative, and manage peer relationships [39].

Cancer can delay or even completely derail educational or career goals, in part, due to changes in cognitive or physical function [25]. The AYA HOPE study found that more than half of AYAs reported problems with employment or school after diagnosis [34]. This, in turn, can limit their ability to reach financial autonomy, thus further increasing their dependence on family [15,40,41]. These young adults may experience a reduced sense of identity if they are not able to work or participate in a vocation [41]. 

From a legal standpoint, the diagnosis of cancer may bring to light the need for advanced care planning, including the designation of guardianship or medical power of attorney. Although a patient 18 years or older is granted the legal ability to make their own medical decisions, the reality is that many of these patients will still want to have their caregiver’s input for such decisions [42]. For AYA parents of young children, they may feel the need to seek legal counsel to help with guardianship/custody arrangements and financial planning. 

With regards to sexual health, AYA patients with cancer may experience disruption/delay of puberty, sexual dysfunction, and infertility. This may further derail plans for having children. Sexuality, in the social sense, is also adversely affected; many of these patients have difficulty establishing their sexual identity due to the disruption in development, the physical changes and limitations of their cancer, concerns with body image, and/or anxiety/depression that further suppresses libido [18,34,43].

The last two decades have seen an increase in literature elucidating the social needs of AYA cancer patients. Studies have identified social isolation [37,43,44], educational/ vocational counseling [41,44], sexual health [34,37,44], fertility [8,18,37,44], and financial burden [34] as areas of unmet needs in AYA patients. However, there is a paucity of data on evidence-based interventions. Subsequent research is needed to evaluate the effect of these interventions on short- and long-term outcomes.

III. Spiritual Pain

Spiritual pain is also prevalent among adolescents and young adults with cancer and can have a negative effect on their mental health [34]. They may experience distress as they are forced to confront their mortality in a phase of life normally characterized by perceptions of invisibility [45]. These individuals may grieve over an unlived life or unrealized goals, which may leave them questioning the meaning of their lives. AYA parents may also feel deep spiritual distress related to the fear of not fulfilling their responsibilities as parents [46]. Many older adults have experienced loss in various forms but through experience, acceptance, and spirituality, they can find meaning and hope, thereby positively influencing their ability to cope [47]. These young adults have not had the benefit of time and experience to mature their coping skills [48]. Addressing the spiritual needs of AYAs with cancer can have a positive impact on wellbeing although the literature is not conclusive [49].

### 3.2. Resilience and Posttraumatic Growth

Although the focus of this review is the psychological distress in AYAs with cancer, it is important to note that the experience of cancer can lead to resilience and personal growth. In the AYA HOPE study, AYAs reported a positive impact from their cancer experience on relationships, future plans/goal setting, spirituality/religious beliefs, and health competence [50]. Similarly, adolescent cancer patients have reported levels of self-esteem and hopefulness comparable to health controls, supporting previous conclusions that adolescents can cope effectively, think positively, and establish hopefulness [51]. Monteiro et al. found that emotional distress did not differ significantly between AYA cancer patients, AYA cancer survivors, and controls, although AYA cancer participants did experience lower QOL and personal growth compared to controls [52]. One study found that their perceptions of health-related quality of life (HRQOL) issues also tend to be more positive when compared to health care professionals; health care professionals rated AYAs’ physical symptoms higher than AYAs while AYAs rated overall physical health, QOL, happiness, satisfaction, usefulness, and support from others significantly higher than health care professionals [53]. 

The concept of posttraumatic growth has been described as the positive psychological change that develops after traumatic life events while resilience is the ability to cope with negative experiences while maintaining normal function/capacity [54]. A majority of childhood and adult cancer survivors experience posttraumatic growth [55,56]; however, in AYAs, a longitudinal study by Husson et al. showed that posttraumatic growth is dynamic and could change over time [57]. Rosenberg et al. further explored resilience in AYAs with cancer, finding that AYAs defined resilience as the ability to handle adversity and associated their resilience with their personal resources and learnable skills in the areas of stress management, goal setting, positive reframing, and benefit finding [58]. The follow-up pilot study aimed to determine the feasibility of a resilience-building intervention for AYAs with serious illness, called the Promoting Resilience in Stress Management (PRISM) intervention [59]. The authors found that the study was feasible and although the study was not designed nor powered to evaluate efficacy, exploratory analyses did not find statistically significant changes in patient-reported resilience [59]. 

Both resilience and posttraumatic growth were positively associated with satisfaction in life and health-related QOL, thus highlighting these as areas of future research in order to improve short- and long-term psychological outcomes for AYAs. Conversations surrounding the construct of hope have been suggested as a possible intervention.

## 4. Conclusions

AYAs with cancer are a particularly vulnerable population that is unique from their younger and older counterparts. They experience a significant psychological, social, and spiritual pain burden due to cancer’s interruption of normal development and the transition to independence. Although a concerted effort to increase the knowledge about the AYA population and their unique needs has been made, there are still many gaps in knowledge and care delivery. Future research should focus on patient factors that portend higher risk for psychological distress, early identification of those at higher risk for psychological distress, additional validated tools for measuring AYA needs, and evidence-based interventions to support their psychological, emotional, and social health. Special attention should be placed on interventions that address vocational, financial, and sexual needs as these significantly impact psychological wellbeing. Interventions that promote resilience and posttraumatic growth have also emerged as possible future directions. 

While additional research will help further the knowledge base to better assess and intervene in AYA needs, thereby minimizing suffering and improving quality of life, oncology programs around the country and worldwide are increasingly recognizing the need for tailored AYA programs. Separate clinical spaces, including clinic and inpatient units, and personnel that are specifically trained for working with AYA patients have been proposed as areas of improvement [8,18,37]. These support systems may help address unmet needs for AYAs that may otherwise be missed in traditional pediatric or adult oncology programs and are also areas of future research and growth.
